# Identification of Patterns of Hospitalizations in Child and Adolescent Mental Health Service

**DOI:** 10.1007/s11414-024-09887-2

**Published:** 2024-06-06

**Authors:** Sébastien Urben, Philippe Golay, Alberto Forte, Swen Courousse, Carole Kapp, Kerstin Jessica Plessen, Marco Armando

**Affiliations:** 1 Division of Child and Adolescent Psychiatry, Department of Psychiatry, Lausanne University Hospital (CHUV), Lausanne, Switzerland; 2 Faculty of Biology and Medicine, University of Lausanne, Lausanne, Switzerland; 3 Service of Community Psychiatry, Department of Psychiatry, Lausanne University Hospital and University of Lausanne, Lausanne, Switzerland; 4 Service of General Psychiatry, Lausanne University Hospital and University of Lausanne, Lausanne, Switzerland

**Keywords:** Mental health, Adolescents, Hospitalization, Cluster analysis

## Abstract

Adolescence is a time of increased vulnerability to mental health conditions, which may necessitate hospitalization. This study sought to identify and characterize patterns of adolescent (re-)hospitalizations. The one-year (re-)hospitalization patterns of 233 adolescents were analyzed. The sequences of hospitalization and discharge was examined using cluster analyses. Results revealed five distinct (re-)hospitalization patterns or clusters: Cluster A represented brief hospitalizations with 56 cases (24.03%) averaging 7.71 days; cluster B consisted of repetitive short hospitalizations involving 97 cases (41.63%) with an average of 19.90 days; cluster C encompassed repetitive medium hospitalizations included 66 cases (28.33%) averaging 41.33 days; cluster D included long hospitalizations with 11 cases (4.72%) and an average of 99.36 days; cluster E depicted chronic hospitalizations, accounting for 3 cases (1.29%) with an average stay of 138.67 days. Despite no age-based differences across clusters, distinctions were noted in terms of sex, diagnoses, and severity of clinical and psychosocial difficulties. The study identified characteristics of both regular and atypical adolescent hospitalization users, emphasizing the distribution of hospitalization days and their associated clinical attributes. Such insights are pivotal for enhancing the organization of child and adolescent mental health services to cater to the growing care requirements of this age group.

## Introduction

Adolescence is an important developmental period as a precursor to a fulfilling, healthy, and balanced adult life [1]. However, many psychological and physiological changes take place during adolescence, heightening vulnerability to social challenges that can foster social insecurity and potential disorientation [2]. Indeed, the vast majority (75%) of mental health problems manifest before the age of 18, [3, 4] and approximately 40% of these young individuals continue to require care into adulthood [5, 6]. Thus, early intervention for mental health problems in adolescence represent a unique window of opportunity to alleviate future difficulties [7]. In this sense, different types of interventions, according to the clinical staging model, can be proposed depending on the status of the adolescent in distress. From this viewpoint, inpatient treatments represent one of the possible interventions, especially with the goal of treating and stabilizing acute symptomatology. [8, 9]

The goal of hospitalization is to alleviate symptoms and mitigate risks, [8, 10] ultimately promoting a functional level conducive to less intensive care or rehabilitation [11–13]. However, the resources for inpatient care, both in terms of public health and human capacity, are notably constrained [14]. Moreover, the hospital experience can be potentially traumatic for adolescents and can also carry the added burden or “side effect” of stigmatization [15]. However, no previous studies examined the pattern of adolescent (re-)hospitalization in the context of child and adolescent mental health service contrary to adult psychiatry or other medical conditions (for a review see [16]).

### The current study

The main objective of this study is, thus, to examine the (re-)hospitalization patterns among adolescents within child and adolescent mental health service (CAMHS). Specifically, the authors aims to pinpoint distinct groups or “clusters” of adolescents with similar (re-)hospitalization patterns and examine their sociodemographic (e.g., age and sex) and clinical (e.g., diagnoses and the severity of psychosocial symptoms) characteristics.

In particular, the primary objective of this study revolves around the identification of clusters of type of inpatient (multiple) stays. The secondary objective is to examine the differences between the identified clusters in terms of sociodemographic and clinical characteristics.

By doing so, the authors hope to match resource utilization with patient profiles, ultimately refining care need. Such insight is not only academically enriching but also have clinical implications (i.e., provide the best available care for the patients who need at most) as well as for steering the clinical governance of CAMHS.

## Methods

### Sample

All adolescents aged 13 to 18 admitted to the psychiatric inpatient unit of the Division of Child and Adolescent Psychiatry in Lausanne at the University Hospital between 2019 and 2021 were eligible to take part in the study. Most of these patients exhibited severe behavioral or emotional challenges, often accompanied by acute suicidal ideations or behaviors, such as suicide attempts. The main objective of their hospitalization was to alleviate symptoms to a level where outpatient care, like day-care centers or ambulatory treatment, would suffice. In the unit, a multidisciplinary team, including child and adolescent psychiatrists, nurses, and pediatricians, attended to the patients.

The dataset comprised 489 inpatient stays, representing 357 distinct patients (with a single patient having up to six recorded inpatient stays). The information includes hospitalization dates, basic available sociodemographic data (age and sex), and clinical characteristics (diagnosis as well as clinical and psychosocial difficulties) from institutional medical records. The study was approved by the Ethics Committee of the Vaud Canton (#2022–01217).

### Measures

The age, sex, and the International Classification of Diseases (ICD) diagnosis has been extracted. In addition, clinicians consistently employed the Health of the Nation Outcome Scales for Children and Adolescents (HoNOSCA [17–19]) to assess the severity of clinical and psychosocial difficulties. The HoNOSCA consists of 15 items, with each scored on a 5-point Likert scale ranging from 0 (*no problem*) to 4 (*severe to very severe problem*). In the current study, only the initial 13 items were used, as they pertained directly to patient difficulties, while the last two items did not. Notably, the reliability of these final two items has been questioned [20]. The total score represented the severity of clinical and psychosocial difficulties, with higher scores indicating pronounced difficulties. The HoNOSCA has been rated by trained clinicians with the context of clinical routine assessment. Clinicians used all the available source of information (from youths, parents, other health professional) to rate the items of the HoNOSCA.

### Data analyses

The analytical methods mirrored those previously implemented in an adult psychiatry study [21]. Analyses were performed using the R software [22]. First, to address the main objective (i.e., identifying patterns of inpatient use), the authors used the TraMineR package [23] allowing the analysis of discrete event sequences, specifically hospitalization dates spanning a year. These sequences were treated as states. By using the “optimal matching algorithm,” the authors calculated the dissimilarity for each pair of sequences. This algorithm determines edit distances that reflect the least-costly means of transforming one sequence into another through insertions, deletions, or substitutions [24]. It enabled the calculation of a dissimilarity matrix for each patient. Subsequently, the authors computed a cluster analysis on this matrix employing the Ward method [25]. The criteria for identifying clusters were interpretability, the number of prototypical sequences, and the size of the clusters. The clusters were interpreted using visualization and statistical tools that enabled us to extract one or several sequences deemed statistically representative of the entire group. [26]

To address the second objective, which aimed to assess differences in sociodemographic and clinical characteristics between patterns, the authors used a Bayesian statistical approach. This approach offers a solution to the multiple comparisons dilemma [27–29]. Specifically, the first model, the homogenous one (A = B = C = D = E), posited that the five groups were undifferentiated. Conversely, the heterogeneous model (A ≠ B ≠ C ≠ D ≠ E) posited complete differentiation among all groups. The authors examined all intermediate possibilities to identify the most probable and fitting model. For this purpose, the authors employed the Bayes R2STATS group models calculator. [30]

## Results

The authors adjusted the data to initiate each sequence with the patient's first hospitalization. Then, the patterns of (re-)hospitalizations were followed over a 1-year period. Consequently, based on the 357 distinct patients, the authors analyzed the data of 233 patients for whom a full year of follow-up data are available.

Within this group, there were a total of 6,522 hospitalization days. The average age of patients was 15.73 years (*SD* = 1.15), and 65.7% (*n* = 153) were female. The ICD diagnoses were distributed as follows: 10.4% (*n* = 24) had mental disorders due to the use of psychoactive substances (F1.x); 10.0% (*n* = 23) had schizophrenia spectrum disorders (F2.x); 32.2% (*n* = 74) exhibited mood (affective) disorders (F3.x); 33.5% (*n* = 77) presented with anxiety or obsessive–compulsive disorders (F4.x); 0.9% (*n* = 2) showed eating and sleeping disorders (F5.x); 2.6% (*n* = 6) were diagnosed with disorders of personality (F6.x); 0.9% (*n* = 2) had intellectual disabilities (F7.x); 1.3% (*n* = 3) showed disorders of psychological development (F84.9);and, finally, 8.3% (*n* = 19) had behavioral and emotional disorders (F9.x).

**Figure 1:**
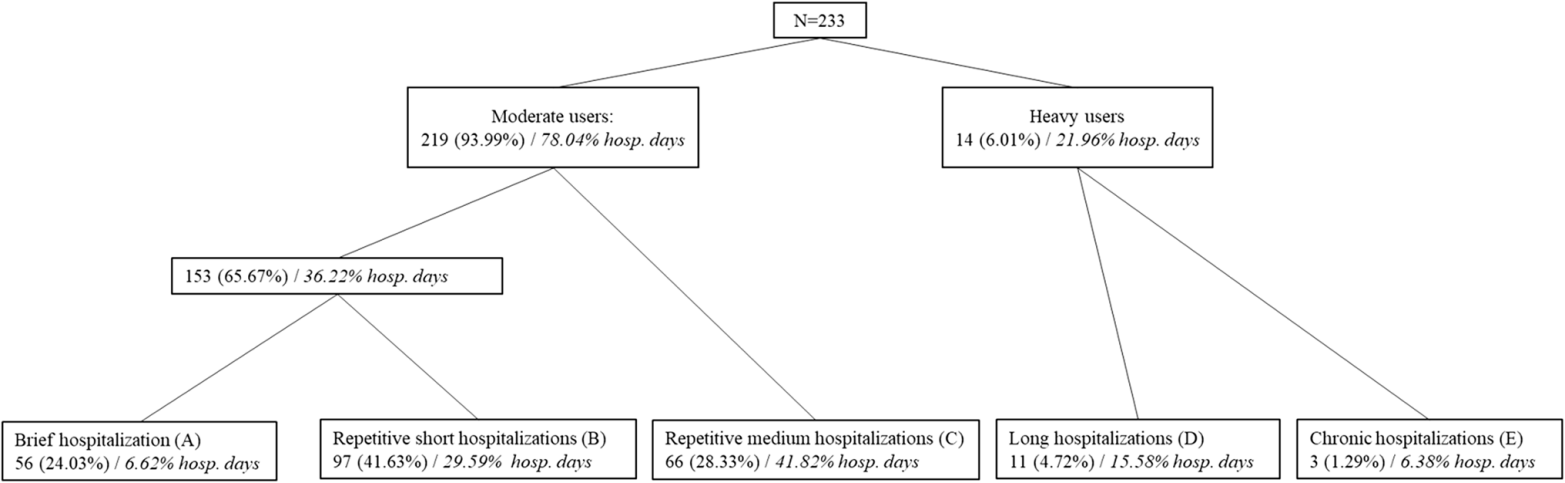
Clusters among 1-year hospitalizations trajectory. *Note.* Name of the clusters, number of patients (% of the total number of patients). Hosp. days, hospitalizations days

Upon segmenting the data into an increasing number of clusters, the authors identified five meaningful clusters (Fig. 1). An attempt to extract a sixth cluster did not yield further clarity or distinction in the usage patterns. Consequently, the authors settled on a five-cluster solution, detailed below.

Initially, the patients may be distinguished between the regular “moderate” users, accounting for 93.99% (*n* = 219) of the sample, and the atypical “heavy” users, making up 6.01% (*n* = 14; Fig. 1). Notably, the “moderate” users represented 78.04% of total hospitalization days, whereas the “heavy” users represented the remaining 21.96%. Approximately 20% of the number of hospitalization days are allocated to only 6% of the patients. On the opposite end of the spectrum, patients with brief hospitalizations (24%) refer to only about 6% of total hospitalization days. Within the regular “moderate” user category, three primary clusters were discerned: A. Brief hospitalization comprising 24.03% (*n* = 56) of users with an average duration (*M*_*days*_) of 7.71 days; B. Repetitive short hospitalizations consisting of 41.63% (*n* = 97) of users with *M*_*days*_ = 19.90; C. Repetitive medium hospitalizations representing 28.33% (*n* = 66) of users with an average stay of *M*_*days*_ = 41.33. Among the atypical users, the authors identified two clusters: D. Long hospitalizations: 4.72% (*n* = 11) with a hospitalization duration averaging *M*_*days*_ = 99.36; E. Chronic hospitalizations 1.29% (*n* = 3) whose stays averaged *M*_*days*_ = 138.67.

**Figure 2:**
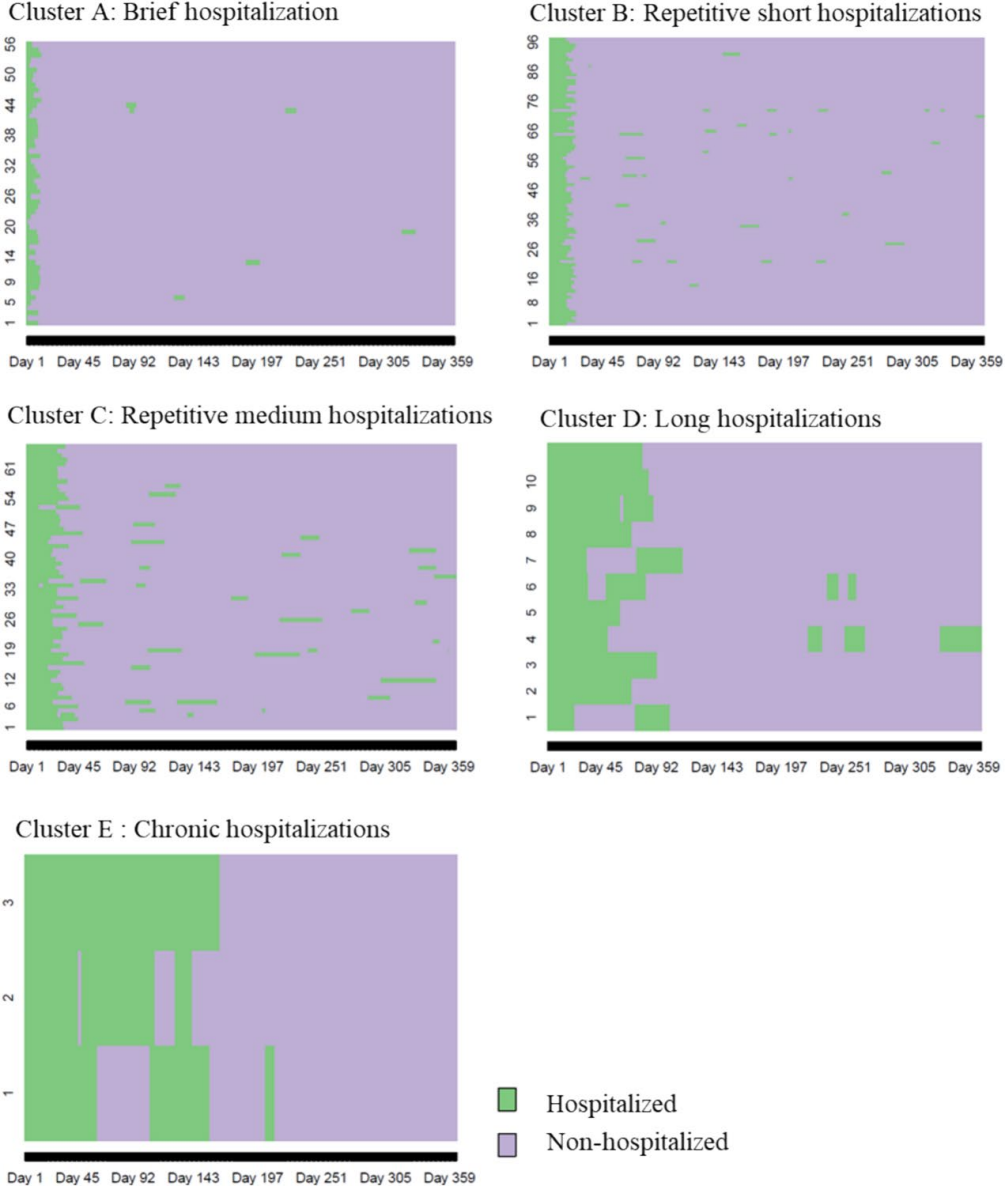
Clusters’ description

Each cluster represents a unique pattern. In particular, Cluster A represented brief hospitalizations, primarily singular events, Cluster B was characterized by repetitive re-hospitalizations of short duration, Cluster C referred to frequent re-hospitalization of medium-term duration, Cluster D corresponded to multiple extended hospital stays, whereas as Cluster E was indicative of chronic hospitalization of long-term duration (Fig. 2).

Bayesian statistical analyses suggested significant differences in the number of hospitalization days across these clusters (Table 1). A consistent increase in service use was observed from cluster A to cluster E.

**Table 1:** Clusters characteristics in terms of number of patients and total duration of hospitalization

	Clusters						
Days	A: Brief hospitalization	B: Repetitive short hospitalizations	C: Repetitive medium hospitalizations	D: Long hospitalizations	E: Chronic hospitalizations	Total	Bayesian statistics
Mean	7.71	19.90	41.33	92.36	138.67	27.99	(A ≠ B ≠ C ≠ D ≠ E)
SD	4.68	8.29	18.53	62.36	24.11	28.89	
Min	0	13	24	50	116	0	
Max	19	84	119	277	164	277	
# patients	56	97	66	11	3	233	
%patients	24.03	41.63	28.33	4.72	1.29	100	
%hosp. days	6.62	29.59	41.82	15.58	6.38	100	

To better understand the distinctions among the clusters, the differences between them in terms of age, sex, diagnosis and symptom severity were evaluated (Table 2). The results did not reveal any differences in terms of age. However, there were notable differences in the gender distributions: clusters A (brief hosp.), B (repetitive short hosp.), and C (repetitive medium hosp.) had a higher proportion of girls, while boys were more predominant in clusters D (long hosp.) and E (chronic hosp.). It is important to note that clusters D (long hosp.) and E (chronic hosp.) together comprised only 14 patients. Moreover, the authors observed differences in terms of diagnosis between the clusters. In particular, youths from cluster A (brief hosp.) had mainly anxiety or obsessive–compulsive disorders, whereas those in clusters B (repetitive short hosp.), C (repetitive medium hosp.), D (long hosp.), and E (chronic hosp.) demonstrated a more varied diagnostic profile. In addition, youths from clusters A (brief hosp.) and B (repetitive short hosp.) exhibited milder symptoms and fewer psychosocial difficulties, as reflected by their lower HoNOSCA scores. Conversely, youths in clusters C (repetitive medium hosp.), D (long hosp.), and E (chronic hosp.) presented with more severe symptoms and greater psychosocial difficulties.

**Table 2:** Socio-demographic and clinical characteristics by clusters

		Clusters					
		A: Brief hospitalization	B: Repetitive short hospitalizations	C: Repetitive medium hospitalizations	D: Long hospitalizations	E: Chronic hospitalizations	Bayesian statistics
Age	Years	15.82 (1.11)	15.67 (1.08)	15.65 (1.29)	16.09 (1.22)	16.00 (0.00)	(A = B = C = D = E)
Sex	% Girls	67.9	70.1	63.6	36.4	33.3	(A = B = C) ≠ (D = E)
Diagnostics	F1.x	7.5	8.2	15.2	18.2	0.0	(A) ≠ (B = C = D = E)
	F2.x	0.0	6.2	19.7	27.3	33.3	
	F3.x	22.6	38.1	33.3	27.3	0.0	
	F4.x	52.8	32.0	22.7	9.1	66.7	
	F5.x	0.0	0.0	1.5	9.1	0.0	
	F6.x	1.9	4.1	0.0	9.1	0.0	
	F7.x	1.9	0.0	1.5	0.0	0.0	
	F84.8	0.0	2.1	1.5	0.0	0.0	
	F9.x	13.2	9.3	4.5	0.0	0.0	
HoNOSCA		18.45 (7.95)	20.64 (7.65)	23.98 (6.15)	24.88 (8.20)	26.00 (0.00)	(A = B) ≠ (C = D = E)

Data expressed as mean (SD) or percentage as appropriate. *F1.x*, mental disorders due to the use of psychoactive substances; *F2.x*, schizophrenia spectrum disorders; *F3.x*, mood (affective) disorders; *F4.x*, anxiety or obsessive–compulsive disorders; *F5.x*, eating and sleeping disorders; *F6.x*, disorders of personality; *F7.x*, intellectual disabilities; *F84.9*, disorders of psychological development; *F9.x*, behavioral and emotional disorders

## Discussion

Similar (re-)hospitalization patterns were identified and their sociodemographic and clinical characteristics were examined. Five distinct clusters emerged from the analysis: A: Brief hospitalization; B: Repetitive short hospitalizations; C: Repetitive medium hospitalizations; D: Long hospitalizations; E: Chronic hospitalizations. Although the duration and frequency of hospitalizations varied across clusters, the age of patients did not vary significantly among the clusters. Moreover, there were noticeable differences in terms of gender, diagnoses, and the extent of symptoms severity and psychosocial challenges faced by the patients.

### Patterns of (re-)hospitalization and service organization

The identified clusters in this study are largely consistent with those from previous research examining similar patterns or trajectories in adults psychiatry [21, 31, 32]. Indeed, the ratio between the number of patients and the hospitalization days (a small number of patients necessitate a large number of hospitalization is similar to what was observed in adult psychiatry, where this pattern were observed to be even more pronounced in adult psychiatry where roughly 5% of patients consume a total of 30% of the resources) [21]. Thus, the most severe and chronic cases which represent few adolescents need the most hospitalization days (or resources).

This may be understood in the light of the “stepped care” approach [33–35]. It tailors treatment responses to the patient's clinical stage and needs [36, 37] and may guide the organization of the hospitalization. In that sense, the efficacy of repetitive short stays is often debated, especially when considering the potential adverse effects of hospitalizations [15]. If CAMHS organizations were structured differently, there could be a bolstering of intermediate or outpatient structures (e.g., intensive community treatment, short-stay day care centers, and specialized short-term inpatient crisis units) to prevent such re-hospitalizations. Such structures aim to decrease the need for hospitalizations for adolescents while supporting continuity of care and offering them the most appropriate treatment to sustain their ongoing development. According to this approach, only patients with the most severe difficulties should be hospitalized. Thus, it emphasizes the relevant or “smarter” utilization or “smarter medicine” [38] of inpatient facilities, underscoring the importance of comprehending trends.

### Sociodemographic variables related to the different patterns of (re-)hospitalization

In the analyses of the five clusters, results revealed no age-based differences, a finding consistent with a study on the adult population using analog procedure [21]. More generally, a recent systematic review of the literature [39] yielded inconsistent results. Some studies within this review [39] associated a higher risk of re-hospitalization with younger age, while others found the opposite trend. In another study with a mixed-age population (ranging from 15 to 64 y.o [40]), younger age was found to be predictive of an increased risk of the repetitive medium hospitalization phenomenon.

In the current study, females were overrepresented in the typical user clusters and underrepresented the atypical user. This is somewhat aligned with findings from a systematic review [39], where a majority of studies found an association between being female and an increased risk of re-hospitalization. As for clusters D (long hosp.) and E (chronic hosp.), the underrepresentation of females might be partially due to a significant proportion of patients in these clusters being diagnosed with schizophrenia spectrum disorders. This diagnosis is more prevalent in males than in females, with the difference being particularly marked up to the age of 25 [41–43]. Moreover, it should be noted that clusters D (long hosp.) and E (chronic hosp.) represent only 14 patients, which could influence these findings. In addition, this discrepancy can be partly attributed to the overrepresentation of females in the sample.

### Distinct clinical profiles of hospitalized patients

To summarize, results revealed three main profiles of hospitalized patients. The first profile, represented by cluster A (brief hospitalization with an average duration of 7.71 days), comprised patients exhibiting acute symptomatology and with mild impairment in psychosocial functioning. For these individuals, a brief hospitalization, typically singular, serves to address the immediate crisis and possibly alleviate the external factors contributing to it. This is supported by the fact that re-hospitalization rarely re-occurred within a year. These individuals, who show clinical benefits from a single hospitalization, are best treated during their acute phases in short-stay hospitalization units which is consistent with the hospitalization’s goal to alleviate symptoms [8, 10] and promote functional level [11–13].

The second profile encompasses patients from clusters B (repetitive short hospitalizations with an average duration across hospitalizations of 19.90 days) and C (repetitive medium hospitalizations with an average of 41.33 days). These patients are marked by their propensity for re-hospitalization, which differs in frequency and length, with the repetitive medium hospitalizations group experiencing more prolonged and frequent stays. Predominantly, this group was diagnosed with mood disorders and displayed subpar psychosocial functioning. These patients together accounted for 70% of the patients and 70% of the hospitalization days. Given the high re-hospitalization rate, the authors assumed that a different management strategy needs to be developed for these patients [44]. Indeed, while traditional hospitalization proved beneficial, these patients consumed a significant portion of the available hospital days.

The third profile, represented by clusters D (long hospitalizations with an average duration of 99.36 days) and E (chronic hospitalizations with an average of 138.67 days), consisted of patients with the most severe diagnoses and the most compromised psychosocial functioning which is consistent with previous findings [45, 46]. Although this group generally benefited from traditional hospitalization, an extended duration was necessary to achieve symptom remission that aligned with outpatient treatment standards. Yet, prolonged hospital stays can introduce adverse consequences such as stigmatization and de-socialization [15]. Given these considerations, re-evaluating the efficacy and suitability of extended hospitalizations, particularly for clusters D and E, becomes paramount. While a hospital environment is undeniably beneficial during acute phases, longer-term treatment might find greater success in social-health facilities aimed at fostering reintegration into the patient's natural social setting. Adopting this approach could also free up critical resources within acute inpatient units for more urgent cases.

### Limitations and future perspectives

This study has several limitations. Firstly, owing to its retrospective design based on clinical data, it offers limited insights into the clinical condition of the patients. While future studies might benefit from a prospective design, the essential attributes (due to retrospective study of data collected in routine clinical care) for clinical characterization of the clinical and socio-demographic variables, such as age, sex, and diagnoses, were the only available information in this research. Additionally, the analysis was confined to a 1-year period. A more extended study might yield additional insights, such as patterns observable over a longer timeframe. It is also worth noting that the data might have been influenced by the impact of the sanitary crisis on youth mental health. Moreover, the authors did not have access to the type of familial, social and/or community support that the youths may benefit after the hospitalization. This may impact the youths’ ability to adhere to treatment recommendations upon discharge and ultimately their ability to access the outpatient services and resources. In future research endeavors, adopting a more comprehensive approach that encompasses various treatment modalities, such as outpatient care, daycare, and assertive community treatment, could be beneficial (see for instance [47]).

## Conclusion

This study identifies five distinct patterns of re-hospitalization which varies in terms of socio-demographic variables as well as of clinical characteristics. In particular, results revealed three main profiles of patients’ characteristics related to their need of (re-)hospitalization. Further studies should involve a deeper exploration of these hospitalization patterns and beyond by including other type of treatment as well as by incorporating a broader range of sociodemographic and clinical characteristics. Such research endeavors can help identify patients who may require extended stays or utilize more clinical resources, enabling tailored clinical settings to offer optimal care.

## Implications for Behavioral Health

In this study, results revealed at least three profiles of hospitalized patients according to the number of re-hospitalization and their duration. In this sense, the authors observed that the most severe and chronic cases, which represent a small percentage of the total number of hospitalized adolescents, need the most days of hospitalization. This clearly shows how the needs in terms of therapeutic interventions can be extremely variable in hospitalized patients.

Accordingly, the early identification of the distinct clinical profiles may help to facilitate patient’s trajectory within the available care and, thus, may reduce their distress and optimize the best clinical and functional outcomes. This would clearly have a positive effect not only on the adolescent but also on his family context, which often experiences hospitalization as something traumatic. Moreover, at a health care system level, this would represent an opportunity to optimize the allocation of resources in accordance with the real needs and to articulate the different care options. Thus, the identification of different hospitalization profiles leads to a number of implications for the behavioral health. First, recognizing different profiles of hospitalized patients allows healthcare providers to develop personalized treatment plans. By understanding that some cases are more severe and chronic, resources can be allocated appropriately, and tailor interventions can be developed to meet the specific needs of each patient. Second, by identifying a small percentage of patients require a disproportionate amount of hospitalization days suggests that resources should be allocated strategically. This might involve ensuring sufficient staffing, specialized care, and support services for these more complex cases. Third, by requiring extended hospital stays, long-term care planning becomes crucial. This could involve coordination with community resources, rehabilitation services, and specialists to ensure continuity of care beyond the hospital settings. Four, patients with a history of re-hospitalization may require closer monitoring and follow-up care to prevent future readmissions. This might involve regular follow-ups and mental health and social support to address underlying issues contributing to repeated hospitalizations. Five, by providing education to patients and their families about the variability in therapeutic needs among hospitalized patients can help manage expectations and empower them to be proactive in their care. Understanding the potential for longer hospital stays in certain cases can also facilitate better decision-making and coping strategies.

Thus, the current results help to think the personalized need of the patients and the corresponding organization of CAMHS to address their specific difficulties.

## Data Availability

The data that support the findings of this study are not publicly available due to data protection of the participants, but may be available from the corresponding author upon reasonable request.
